# Regulatory Roles of *SREBF1* and *SREBF2* in Lipid Metabolism and Deposition in Two Chinese Representative Fat-Tailed Sheep Breeds

**DOI:** 10.3390/ani10081317

**Published:** 2020-07-30

**Authors:** Chen Liang, Liying Qiao, Yongli Han, Jianhua Liu, Jianhai Zhang, Wenzhong Liu

**Affiliations:** 1 College of Animal Science, Shanxi Agricultural University, Taigu 030801, Shanxi, China; liangchen@sxau.edu.cn (C.L.); liyingqiao@sxau.edu.cn (L.Q.); ljhbeth@sxau.edu.cn (J.L.);; 2 College of Veterinary Medicine, Shanxi Agricultural University, Taigu 030801, Shanxi, China; yonglihan@163.com (Y.H.); Jianhaiz@sxau.edu.cn (J.Z.)

**Keywords:** *SREBF1*/*2*, fat-tailed sheep, lipid metabolism, adipose tissues

## Abstract

**Simple Summary:**

Sterol regulatory element binding proteins (SREBPs) play the crucial role in regulating the cholesterol and fatty acid metabolism. However, it is unclear whether SREBPs are involved in the regulation of lipid metabolism in fat-tailed sheep. This study reveals the expression profiles of *SREBF1* and *SREBF2* in liver and adipose tissues of two Chinese representative fat-tailed sheep breeds, and provides a new insight for the regulatory role of SREBP1 and SREBP2 in fat metabolism and deposition in fat-tailed sheep.

**Abstract:**

Sterol regulatory element binding proteins (SREBPs) can regulate the lipid homeostasis by regulating its target genes, which are crucial for the cholesterol and fatty acid metabolism. However, the transcriptional regulation role of SREBPs in fat-tailed sheep is unclear. In this study, two Chinese representative breeds of total 80 fat-tailed sheep were employed, serum triglyceride, total cholesterol (TC), non-esterified fatty acid (NEFA), high-density lipoprotein cholesterol, low-density lipoprotein cholesterol, and mRNA expressions of SREBF1 and SREBF2 in seven different adipose tissues and liver were examined in sheep at the ages of 4, 6, 8, 10, and 12 months, respectively. The subcellular localization and function of SREBP1/2 were predicted through bioinformatics approaches. The results demonstrated that serum TC and NEFA levels among breeds were significantly different, and most serum indices were dynamically altered in an age-dependent manner. The mRNA expression profiling of *SREBF1* and *SREBF2* are breed-specific with temporal and spatial expressions differences. Further analysis shows that *SREBF1*/*2* transcriptional levels and tail traits are closely related. All investigations simplify that *SREBF1*/*2* play a crucial role in lipid metabolism and deposition during growth and development of the fat-tailed sheep, which also provides a novel insight for revealing the genetic mechanism of different tail type and meat quality.

## 1. Introduction

Domesticated sheep are economically important livestock species, and provide many daily prerequisites for human consumption including meat, milk, and wool worldwide [1]. High-quality domesticated sheep breed is considered as a valuable genetic resource for the global animal husbandry [2]. The fat-tailed sheep is widely bred in China and other countries, due to its high adaptability to different climatic conditions, disease resistance and high production in nutritionally challenging environments [3]. While the tail fat plays an important role in this adaptive process. It has been proved that fatty acid content and lipid deposition are vital for sensory, nutritional, and technological properties of mutton [4]. Lipid metabolism is also an extremely important physiological process maintaining nutrient adjustment, homeostasis, and animal health [5,6]. Thus, it is necessary that exploring the mechanisms of fat metabolism and deposition in fat-tailed sheep.

Sterol regulatory element binding proteins (SREBPs) can regulate the lipid homeostasis by regulating its target genes, which are crucial for the cholesterol and fatty acid metabolism [7,8]. The mature forms of SREBPs, are transcriptionally active and are translocated into the nucleus where they bind to the promoters of SREBPs target genes, are involved in lipid metabolism [9]. For example, ADD1/SREBP1c, which is encoded by *SREBF1*, mainly promotes the fatty acid synthesis by activating many genes, and is involved in lipid metabolism in fat tissue and liver [7]. SREBP2, another member of the SREBPs nuclear transcription factor family, which is encoded by *SREBF2*, activates the target gene transcription and the gene expressions on the cholesterol biosynthesis pathway by binding to the sterol regulator and promoter/enhancer in the lipid synthetic enzyme gene [10,11]. However, the role of the transcriptional regulation of SREBPs in fat-tailed sheep is unclear.

Therefore, we hypothesized that the expression patterns of *SREBF1* and *SREBF2* in liver and adipose tissues of two representative Chinese fat-tailed sheep breeds are different in temporal and spatial levels. Furthermore, we assume that serum lipid metabolism indices including triglyceride (TG), total cholesterol (TC), non-esterified fatty acid (NEFA), high-density lipoprotein cholesterol (HDLC), and low-density lipoprotein cholesterol (LDLC) are related with SREBP1/2 expressions. Thus, the objective of this study is to investigate the transcriptional levels of *SREBF1* and *SREBF2* in the various adipose tissues and liver of the fat-tailed sheep with the different development stages, and provide a new insight for revealing the regulation function of SREBP1 and SREBP2 in fat metabolism and deposition in fat-tailed sheep.

## 2. Materials and Methods 

### 2.1. Animals and Ethical Statement

**Figure 1:**
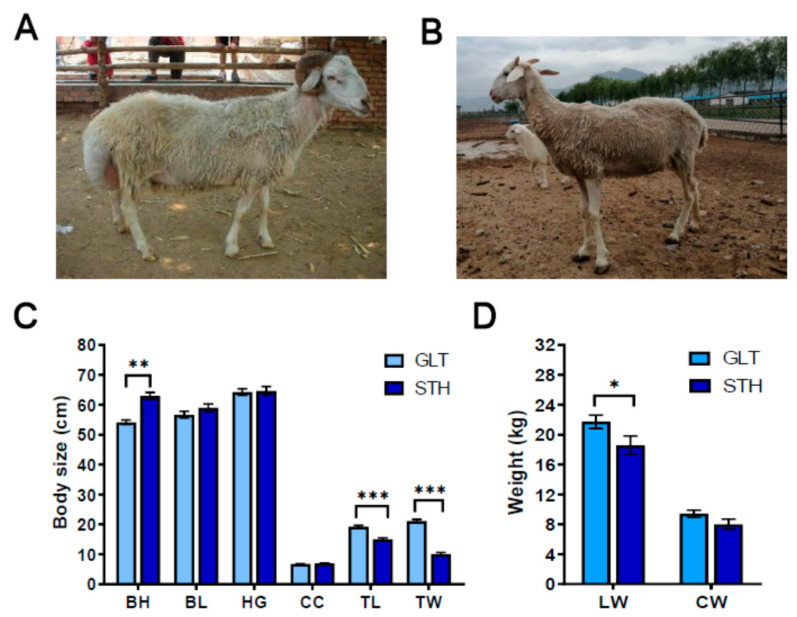
Characters of body size and weight in two Chinese fat-tailed sheep breed. GLT and STH indicate Guangling large tailed sheep and small tailed Han sheep respectively. (**A**) The representative photograph of GLT. (**B**) The representative photograph of STH. (**C**) Comparison of body size parameters including body height (BH), body length (BL), heart girth (HG), cannon circumference (CC), tail length (TL), and width (TW) in GLT and STH, ** *p* < 0.01; *** *p* < 0.001. (**D**) Difference in live weight (LW) and carcass weight (CW) of GLH and STH. * *p* < 0.05.

Two fat-tailed sheep breeds of Guangling large tailed (GLT) and small tailed Han (STH) in China were chose for this study. GLT is native to Guangling County and its surrounding area in the northern part of Shanxi Province. GLT is a famous sheep breed with both meat and fat for long-term selection by Mongolian sheep. STH, is bred from the cross between local big sheep and Xinjiang fine-wool sheep, is one of the best sheep breeds for skin and meat in China. STH has the characteristics of fast growth and development, higher reproductive ability and strong adaptability [12]. GLT is typical large tails and STH is characterized by a small tail. Both of them have better productive performance and the representative photographs of both breeds of sheep were shown in Figure 1.

Total 40 GLTs and 40 STHs sheep including 4, 6, 8, 10, and 12 month of age with a half male or female was selected from two feeding farms located at Shanxi and Shandong Province. The two farms have a similar natural climatic environment and feed management style. All sheep were fed to the same levels diet compositions and nutritional levels. The diet compositions and nutrient levels of sheep consisted of 30.9% corn silage, 44.2% corn, 17% soybean meal, 5% wheat bran, 1% NaCl, 0.3% CaHPO_4_; 0.3% NaHCO_3_, 1% mineral powder, and 0.3% premix (mixed with vitamin, trace elements, and so on). The preparation and management of diets were conducted according to China’s national sheep feeding standards NY/T816-2004. The contents of nutrient levels are: dry matter (DM) 100%, digestible energy (DE) 13.74 MJ/kg, crude protein (CP) 10.69, Ca 5.19 g, P 2.98 g, and so on.

Before 10 days for slaughter, all sheep were transferred and raised on one farm located in Shanxi Agricultural University. The same management condition and diets levels were maintained for adaptation. At 4, 6, 8, 10, and 12 month of age, 8 GLTs and 8 STHs of a half male and female sheep were slaughtered respectively for sample collection. This experiment was conducted according to the guidelines for the care and use of animals for scientific purposes. The protocol was approved by the Institutional Animal Care and Use Ethics Committee of Shanxi Agricultural University (No. 201002001).

### 2.2. Body Measurement

The body size and weight measurement traits of all sheep, i.e., body height at withers (BH), body length (BL), heart girth (HG), cannon circumference (CC), tail length (TL), tail width (TW), live weight (LW), carcass weight (CW), and absolute tail fat weight (ATW) were determined by using measuring tape and platform scale according to the standard procedure. Relative tail fat weight (RTW) and dressing percentage (DP) were calculated as follows: RTW = ATW/CW; DP = CW/LW.

### 2.3. Serum and Tissues Collection

Four males and four females from each breed were randomly selected and slaughtered at 4, 6, 8, 10, and 12 months of age, respectively. Blood samples were collected from an external jugular vein. Blood was allowed to clot, followed by centrifugation at 3000 rpm for 10 min. The serum was collected and sent to measurement of serum biochemical parameters. Adipose tissues, i.e., tail fat (TA), great omental fat (GO), subcutaneous fat (SC), small omentum fat (SO), perirenal fat (PR), retroperitoneal fat (RP), mesenteric fat (MT), and liver (LV), were rapidly dissected, weighed, and placed in liquid nitrogen and stored at −80 °C for further analyses.

### 2.4. Measurement of Serum Biochemical Parameters

Serum samples were separated from all sheep to measure the contents of key biochemical indices related to lipid metabolism and deposition. The indices include triglyceride (TG), total cholesterol (TC), non-esterified fatty acid (NEFA), high-density lipoprotein cholesterol (HDLC), and low-density lipoprotein cholesterol (LDLC). The concrete operations were conducted according to the manufacturer’s manual of kits (Nanjing Jiancheng Bioengineering Institute, China).

### 2.5. RNA Extraction and Real-Time RT-PCR

Total RNA was extracted from the liver and adipose tissue of the tail, subcutaneous, great and small omental, retroperitoneal, mesenteric, and perirenal tissues by Trizol (Invitrogen, Carlsbad, CA, USA) following the manufacturer’s instructions. Then RNA samples were treated with DNase I, followed by standard reverse transcription using SYBR ^®^PrimeScript^TM^ RT-PCR Master Mix Kit. Real-Time qPCR was carried out using the Mx3000P PCR system (Stratagene, La Jolla, CA, USA). The programs for the amplification were set as follows: activation of polymerase at 95 °C for 10 min, followed by 45 cycles of denaturation at 95 °C for 15 s, and annealing/extension at 60 °C for 1 min. The analysis of dissociation curves was always performed after 45 cycles. Primers were designed by Primer 3 Plus (http://www.bioinformatics.nl/cgi-bin/primer3plus/primer3plus.cgi) and checked by Primer-BLAST (https://www.ncbi.nlm.nih.gov/tools/primer-blast/index.cgi?LINK_LOC=BlastHome). β-actin was used as an internal reference control. The primers are listed as followed:

*SREBF1*, F: GAGCTTCGTGGTTTCCAGAG; R: ATCCAGAAGCTGGTGTGTCC;

*SREBF2*, F: TTTGAGGAGGAAGCGAAGAC; R: CCATGTGTACGTCGGAACAG;

*β-actin*, F: GATCATTGCTCCTCCTGAGC; R: ACATCTGCTGGAAGGTGGAC.

### 2.6. Prediction of SREBP1/2 Subcellular Localization and Function

The subcellular localizations of SREBP1/2 were anticipated by the protein subcellular localization prediction tool PSORT II (http://www.genscript.com/tools/psort). The functions of these two proteins were analyzed by the Prot Fun 2.2 Server (http://www.cbs.dtu.dk/services/ProtFun/).

### 2.7. Statistical Analysis

The data of body size measurements, serum biochemical parameters, and relative mRNA expressions were grouped and subjected to statistical analysis by using a *t*-test and a one-way and two-way ANOVA with Tukey’s post-hoc tests for multiple comparisons in GraphPad Prism 8.0 software (San Diego, CA, USA). The potential associations relationships between SREBP1/2 expression levels and considered traits with breed, age, and gender were analyzed by using bivariate correlation process in the SPSS software (version 26.0, IBM, Chicago, IL, USA) Correlation coefficients of *SREBF1*/*2* mRNA expression levels between the different tissues were calculated respectively in the two fat-tailed sheep by the same way.

## 3. Results

### 3.1. Body Size and Weight in the Two Fat-Tailed Breeds

To evaluate the breed characteristics between GLT and STH, the body size and body weight were evaluated, as shown in Figure 1. The mean body height of GLT was higher significantly than that of STH (*p* < 0.01), while the length and width of tail were less than that of GLT (*p* < 0.001). However, no significant differences were observed in body length, heart girth, and cannon circumference (Figure 1C). Although the live weight of STH was slightly lower compared to that of GLT (*p* < 0.05), the total carcass weights of the two breeds had no significant differences (Figure 1D). These differences fully reflected the characteristics of these two breeds, especially in tail fat deposits.

### 3.2. Dynamical Changes of Serum Lipid Metabolism Indices on Fat-Tailed Sheep

To explore the dynamic changes and lipid metabolic differences between GLT and STH, five serum biochemical parameters involved in lipid metabolism were examined between two fat-tailed breeds at different development stages of 4, 6, 8, 10, and 12 months. The results showed that the average of serum TC, and NEFA at five time points were obviously altered, while other parameters had no significant difference between breeds. Interestingly, only serum NEFA in a ewe was significantly higher than that in the rams’ values (Table S1). 

**Figure 2:**
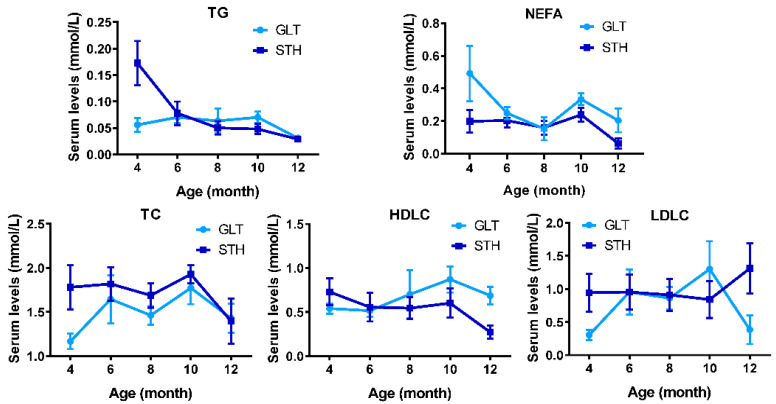
Dynamical changes in five serum lipid metabolism indicators of fat-tailed sheep with age. GLT and STH indicate Guangling large tailed sheep and small tailed Han sheep respectively. TG: triglyceride; TC: total cholesterol; NEFA: non-esterified fatty acid; HDLC: high density lipoprotein cholesterol; LDLC: low density lipoprotein cholesterol.

The changes of these indicators with respect to the age were relatively complicated (Figure 2). In STH, serum TG concentration was the highest at 4 months of age, significantly higher than other months of age, and decreased with the increase of age; TC, NEFA, HDLC, and LDLC concentrations in serum did not alter significantly with an age between 4 and 10 months, but there was a tendency to decrease or increase (LDLC) at the age of 12 months. Comparatively, in GLT, TG did not change significantly between 4 and 12 months. TC, HDLC, and LDLC show similar changes, which firstly increased significantly and then decreased from 4 to 12 months of age. The concentration of NEFA was highest at 4 months and was significantly higher than that at 6 and 8 months and there was a tendency to increase at 10 months and decrease again at 12 months. Those dynamic changes indicate that the lipid metabolism pattern of STH and GLT may be different with developmental ages.

### 3.3. The mRNA Expression Profile of SREBF1 in Liver and Fat Tissues

**Figure 3:**
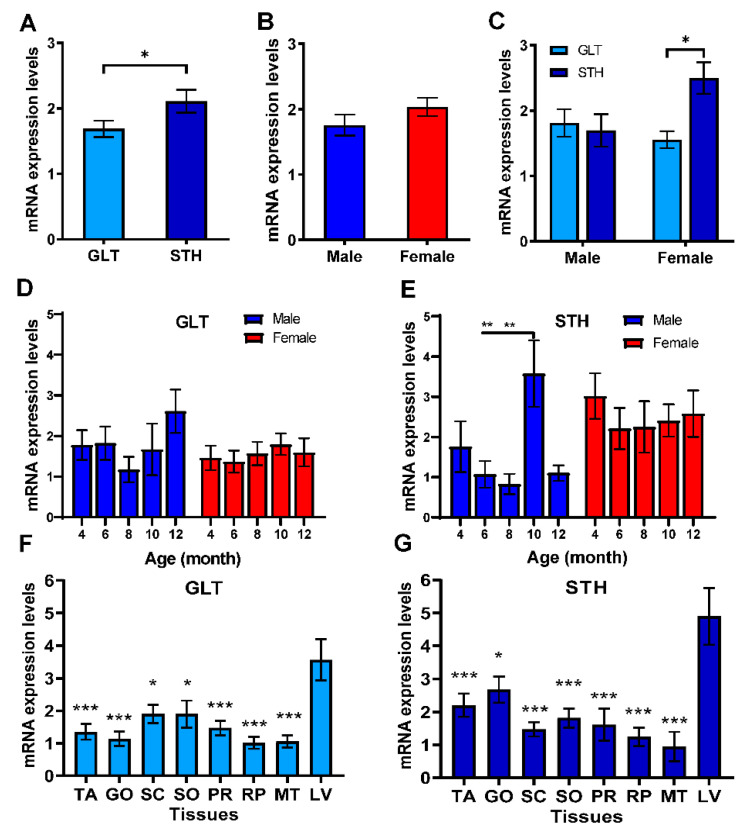
The mRNA expression profiles of *SREBF1* in adipose tissues in two fat-tailed sheep with age by qRT-PCR. (**A**–**C**) The comparisons of global mRNA expression level of *SREBF1* in all tissues of Guangling large tailed sheep (GLT) and small tailed Han sheep (STH) in terms of breed and gender. * *p* < 0.05 indicates the significant differences between GLT and STH. (**D**,**E**) The mean expression levels of SREBF1 in tissues of GLT and STH at the different ages of 4, 6, 8, 10, and 12 months, respectively. * *p* < 0.05, ** *p* < 0.01 indicate the significant differences among ages. (**F**,**G**) Expression abundances of *SREBF1* in tail fat (TA), great omental fat (GO), subcutaneous fat (SC), small omentum fat (SO), perirenal fat (PR), retroperitoneal fat (RP), mesenteric fat (MT), and liver (LV) of GLT and STH. * *p* < 0.05, ** *p* < 0.01 or *** *p* < 0.001 indicate the significant differences compared with liver tissue.

To determinate the role of *SREBF1* in fat metabolism regulation of fat-tailed sheep, the relative mRNA expression profile of SREEP1 in liver and adipose tissues of two breeds at different ages were detected by real-time RT-PCR (qPCR). The result showed that the global mRNA expression was significantly different between GLT and STH (*p* < 0.05, Figure 3A), but no significant gender difference was observed (Figure 3B, Table S2). Merely, in females, the mRNA expression levels in STH were higher than those in GLT (*p* < 0.05, Figure 3C). During the development age, SREBF1 expression was relatively stable in either male or female or combination in GLT (Figure 3D,E). However, the total mRNA expression level was found to be the highest at 10 months of age, and it was significantly higher than that at 8 months of age (*p* < 0.05, Table S2). In male STH, the expression at 10-month was significantly higher than that both at 6- and 8-months (*p* < 0.01, Figure 3E).

We also made an attempt to examine the SREBF1 expression levels in liver and adipose tissues, which are known to be involved in adipogenesis and lipid metabolism. The expression in liver was extremely higher than adipose tissues including tail (TA), great omental (GO), subcutaneous (SC), small omentum (SO), perirenal (PR), retroperitoneal (RP), and mesenteric (MT) fats in both GLT and STH (Figure 3F,G). Similar results were observed in males of GLT and STH. The difference is that the female of GLT did not present a tissue-specific expression, except in MT. In STH females, the expression levels of MT were significantly lower compared to the liver (*p* < 0.05).

Correlation analysis revealed that the mRNA expression levels between liver and GO were significantly positively correlated in GLT (Table S3, r = 0.854, *p* < 0.01). In STH, TA and SC, PR and GO, and PR and SO presented a positive correlation (r = 0.852, 0.915, or 0.979, *p* < 0.05). All results revealed that SREBF1 might play a crucial role in fat metabolism regulation during the growth and development of two breeds of fat-tailed sheep.

### 3.4. mRNA Expression Levels of SREBF2 in Liver and Adipose Tissues

**Figure 4:**
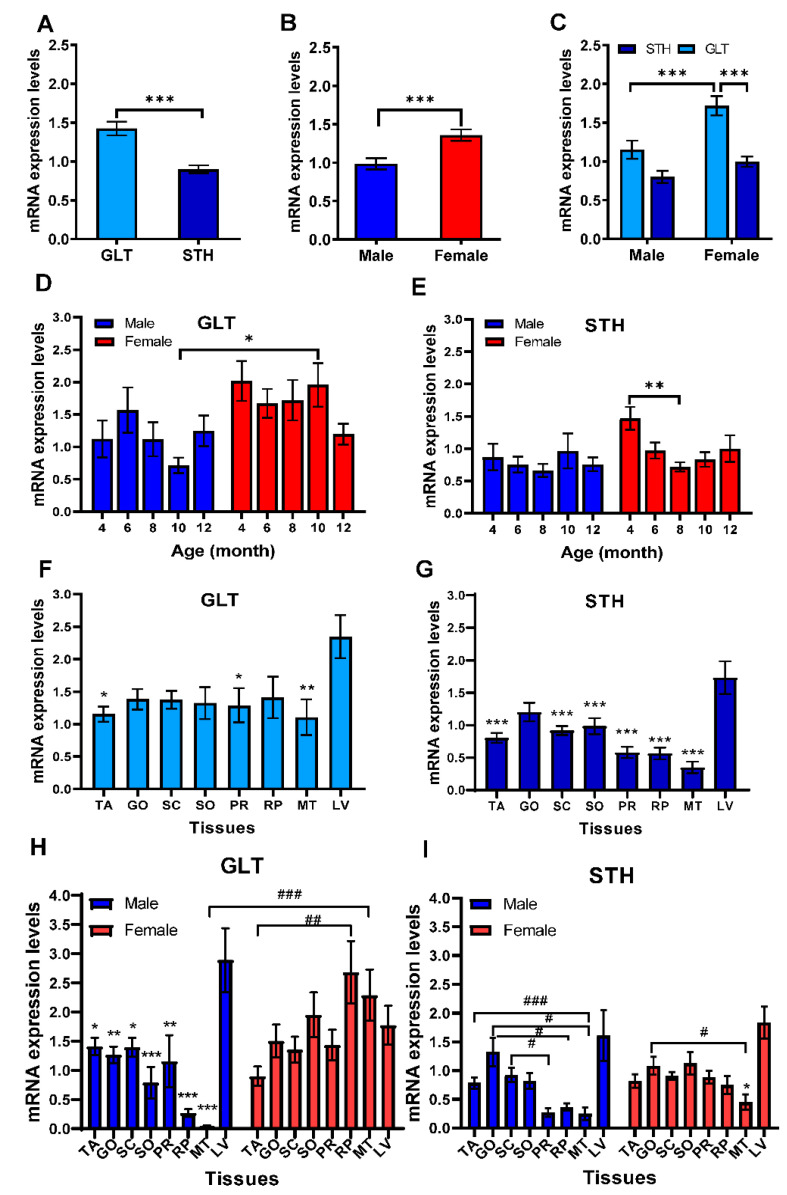
The mRNA expression patterns of *SREBF2* in adipose tissues in two fat-tailed sheep with ages by qRT-PCR. (**A**–**C**) The comparisons of global mRNA expression level of *SREBF2* in all tissues of Guangling large tailed (GLT) and small tailed Han (STH) sheep in terms of breed and gender. * *p* < 0.05 indicates the significant differences between GLT and STH. (**D**,**E**) The mean expression levels of *SREBF2* in tissues of GLT and STH at the different ages of 4, 6, 8, 10, and 12 months, respectively. * *p* < 0.05, ** *p* < 0.01 indicate the significant differences among ages. (**F**–**I**) Expression abundances of *SREBF2* in the tail fat (TA), great omental fat (GO), subcutaneous fat (SC), small omentum fat (SO), perirenal fat (PR), retroperitoneal fat (RP), mesenteric fat (MT), and liver (LV) of male and female sheep. * *p* < 0.05, ** *p* < 0.01 or *** *p* < 0.001 indicate the significant differences compared to the liver. # *p* < 0.05, ## *p* < 0.01, ### *p* < 0.001 show the differences among adipose tissues.

Transcriptional levels of *SREBF2*, another member of the *SREBFs* family, in adipose tissues and liver were further examined and shown in Figure 4. Different from *SREBF1*, *SREBF2* expressed significantly higher in GLT than in STH (*p* < 0.001, Figure 4A) and significantly higher in females than in males (*p* < 0.001, Figure 4B), especially in female of GLT (*p* < 0.001, Figure 4C). The age had no significant effects on the expression in GLT (Figure 4D). However, it was significantly higher at 10-month-old females than in males at the same age in GLT (*p* < 0.05). In STH, it reduced to the lowest point at 8-month-old in comparison to 4-month-old (*p* < 0.05) and increased a little. A similar tendency was observed in female STH (*p* < 0.01, Figure 4E).

Similar to *SREBF1*, the expression of *SREBF2* was enriched in liver than other adipose tissues. In GLT, *SREBF2* mRNA expression levels in liver were significantly higher than TA (*p* < 0.05), PR (*p* < 0.05), and MT (*p* < 0.01, Figure 4F). In STH, *SREBF2* mRNA expressions in liver were markedly higher than all the adipose tissues except GO (*p* < 0.001, Figure 4G). According to the analysis of gender differences, *SREBF2* mRNA expressions in liver of male GLT were significantly higher than all adipose tissues. MT of GLT was the only tissue in which *SREBF2* mRNA expression level differed significantly among male and female (*p* < 0.001). In female GLT, the *SREBF2* mRNA levels were significantly higher in RP than in TA (*p* < 0.01), while there were no significant differences between any other tissues (Figure 4H, Table S4). The correlation coefficients between SC and LV, MT and LV, PR and LV, PR and SC, PR and MT, and SO and MT were significant in GLT (Table S5, *p* < 0.05 or *p* < 0.01).

In male STH, no significant difference was observed in the expression levels of *SREBF2* in the liver, however, significant differences were observed in the expression levels among adipose tissues, such as MT and TA, GO and RP, and SC and PR (Figure 4I). In female STH, the liver showed higher expression levels than any other adipose tissues, and SREBF2 expression in MT was the lowest compared to the liver and GO (*p* < 0.05). Further analysis showed that the significantly positive correlation of *SREBF2* expressions occurred in between GO and SO, PR and GO, PR and SC, and PR and SO in STH (Table S5, *p* < 0.05). These results indicate that *SREBF2* also plays a crucial role in the regulation of lipid metabolism during growth and development of the two breeds of fat-tailed sheep.

### 3.5. Associations between SREBF1/2 Expressions and Slaughter and Tail Traits

Association relationship between *SREBF1*/*2* mRNA expression levels in eight tissues and slaughter and tail traits in sheep were analyzed. The results demonstrated that *SREBF1* mRNA expressions in TA were significantly related to tail-type traits in GLT, such as absolute tail fat weight (ATW) and relative tail fat weight (RTW). The significant correlation was also found between LV and tail length (TL), LV and RTW, and GO and TL, respectively. While in STH, significant correlation was observed only between SREBF1 expressions in PR and body weight (BW), as well as carcass weight (CW), which was not related to tail-type traits (Table 1).

High levels of *SREBF2* mRNA expression in liver were significantly related negatively to tail-type traits including TL, TW, and ATW in GLT. There were also significant negative correlations between SC and TW, as well as between GO and ATW or CW, while the only positive correlation to CW was *SREBF2* mRNA abundances in the SC of STH (Table 2).

### 3.6. Localization and Functional Prediction of SREBP1/2

**Figure 5:**
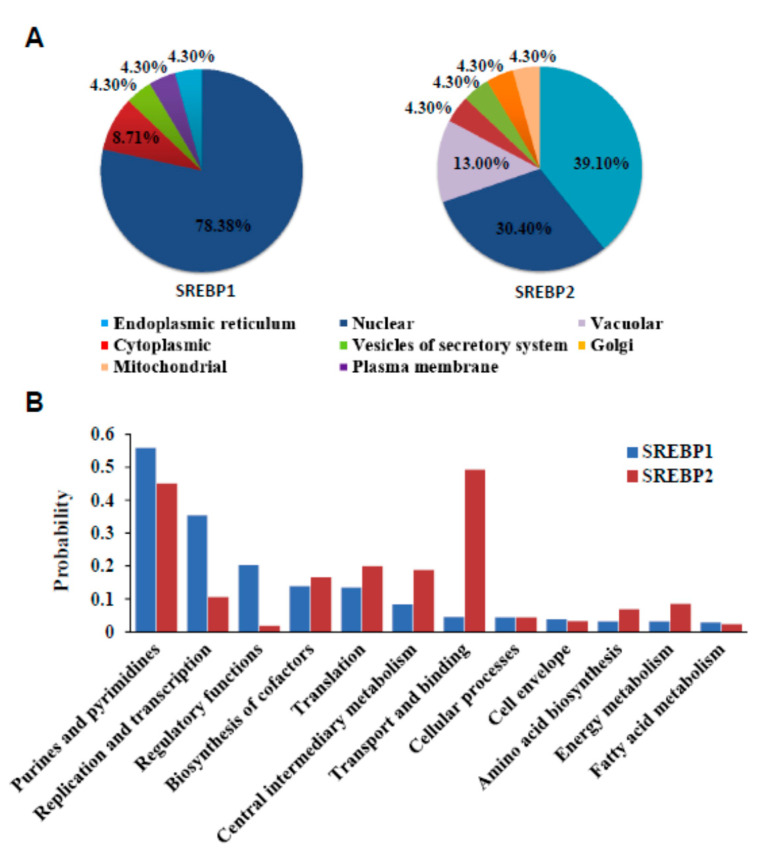
The subcellular localization and predicted function of SREBP1/2 in sheep. (**A**) Subcellular localization of ovine SREBP1 and SREBP2 by contrastive analysis. (**B**) Prediction of functions of SREBP1 and SREBP2 in ovine.

According to SREBP1/2 expression levels in different tissues, age, and breeds, the localization and function of SREBP1/2 in cells were analyzed through bioinformatics approaches. The subcellular locations results showed that SREBP1 and SREBP2 worked mainly in different parts inside cells (Figure 5A). About 78.30% of SREBP1 was distributed in the nucleus, and the rest in the cytoplasm, vesicles of secretory system, plasma membrane, and endoplasmic reticulum. Unlike in SREBP1, only 30.40% of SREBP2 was predicted in the nucleus and 39.1% in the endoplasmic reticulum. In addition, it was also distributed in the vacuole, cytoplasm, and vesicles of the secretory system, Golgi, and mitochondria.

Functional prediction revealed that both SREBP1 and SREBP2 play major roles in many biological processes (Figure 5B). SREBP1 mainly functions in purines and pyrimidines, replication and transcription, and regulatory function, while SREBP2 mainly functions in the process of transport and binding, purines and pyrimidines, translation, and central intermediary metabolism. 

## 4. Discussion

Mutton is becoming attractive and alternative meats on account of the low percentage of fat and good source of essential fatty acids [4,13]. Thus the fat-tailed sheep is raised largely in many countries including China due to its high adaptability to nutritionally challenging environments, disease resistance, and superior meat trait [14]. The tail fat plays a crucial role in maintaining the growth and above merits of sheep [15]. However, excessive tail fat deposition not only consumes more feed but also affects carcass weight and dressing percentage in sheep production [16,17]. So, it is of great significance to elucidate the mechanism of fat metabolism and deposition of fat-tailed sheep and the difference in tail fat size. In the present study, as two typical fat-tailed breeds in China, Guangling large tailed (GLT) and small tailed Han (STH) sheep were selected based on the same feeding management and nutritional conditions. GLT is typical large tails and STH is characterized by a small tail. Both of them have better production performance although the live weight is obviously different during the same period. Our study firstly revealed the dynamical changes of serum lipid metabolism indicators, and the expression patterns of SREBF1 and SREBF2 in liver and adipose tissues of the two sheep breeds.

Serum biochemical indices are closely correlative with production property and the physiological process including nutrient and lipid metabolism adjustment, homeostasis, and sheep health [5,6,18]. Triglyceride (TG) is a fat molecule formed by the condensation of long-chain fatty acids and glycerol. Most tissues can be powered by ATP produced by the oxidative decomposition of fat [19,20]. High density lipoprotein cholesterol (HDLC) and low density lipoprotein cholesterol (LDLC) are the two important parts of the total cholesterol (TC), which affects normal lipid metabolism and resulting in metabolic disorders [21]. As a hydrolyzed product of triglycerides, non-esterified fatty acid (NEFA) is stored in adipose tissue and is also a source of animal energy [22]. In the present study, serum indicators, especially TC and NEFA showed obvious differences and dynamical changes with respect to developmental ages and gender factors. Studies have shown that TC content is positively correlated with sebum rate and liver fat rate [23]. A significant increase in serum NEFA concentration was reported in starved cows [24]. So, these changes in serum biochemical indices implied that the fat metabolism regulation pattern in STH and GLT may be inconsonant. The results may provide the phenotypic basis for the genetic mechanism leading to the difference in the ovine tail type.

Lipid metabolism and deposition is composed of many enzymatic reactions and are often regulated by many molecules [7,25]. Roles of Lpin2/3, angiopoietin-like protein 4, miR-124-3p, and other genes and non-coding RNAs in the regulation of fat deposition from two fat-tailed sheep breeds had been discussed in our previous studies [26,27,28]. Another study by Moradi et al. reported that there is a genetic basis for the phenotypic differences between fat-tailed and thin-tailed sheep [29]. The present study mainly focused on evaluating the crucial role of sterol regulatory element binding proteins (SREBPs) in fat-tailed sheep based on the dynamic alterations observed in serum lipid metabolism indices. SREBPs including SREBP-1a, SREBP-1c, and SREBP2 are mainly responsible for regulating cellular lipogenesis and lipid homeostasis [30]. SREBP-1a and SREBP-1c are encoded by *SREBF1*, which involves in the synthesis of fatty acids and triglyceride, while SREBP2 is encoded by *SREBF2*, which is responsible in regulating the cholesterol gene expression [31]. In this study, both *SREBF1* and *SREBF2* mRNA expression levels in sheep were significantly affected by breed, tissue, and age factors. The expression of the SREBF1 gene in STH is significantly higher than that in GLT, but *SREBF2* gene expression is quiet the opposite. The mRNA expression profiling of *SREBF1* and *SREBF2* are different in the two sheep breeds, which are coincident with previous studies in other fat-tailed sheep [32].

In the comparative study of the lung cells, the expression level of SREBFs on the first day of birth was significantly higher than that of the first 17.5 days of embryonic development [33]. In this study, both *SREBF1* and *SREBF2* expressions in liver and the different adipose tissues were found to be age-dependent (4, 6, 8, 10, and 12 months). For instance, the expression of *SREBF1* in liver was extremely higher than that of those seven adipose tissues, but the difference in *SREBF1* gene expression between two genders was not significant; the interaction of two different factors combination, i.e., between breed and month age, breed and tissue, and tissue and gender also significantly affected its expression. In contrast, the role of *SREBF2* gene was not exactly the same. The expression of *SREBF2* in the liver was also significantly higher than adipose tissue; the expression of female was significantly higher than that of males in GLT. Interestingly, the expression of *SREBF1* gene had spatial–temporal differences, while the expression of *SREBF2* gene did not have this characteristic feature. 

**Figure 6:**
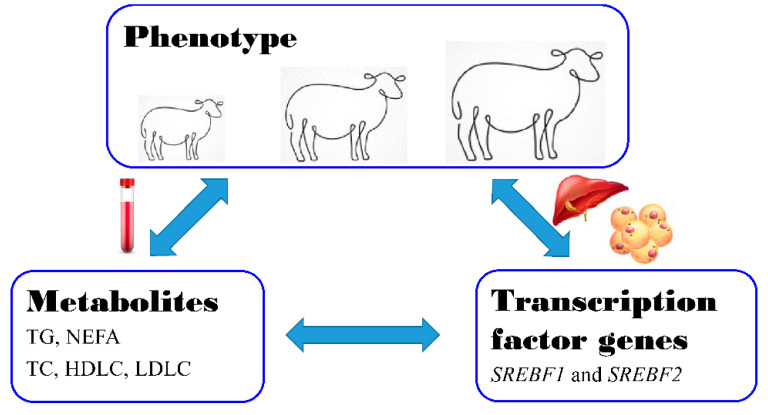
Schematic representation of this study. The role of transcription factor genes *SREBF1* and *SREBF2* in the phenotype of fat deposition and the relationship with metabolites were explored in this study.

In addition, SREBPs are transcriptionally inactive when sterols are abundant, moreover, synthesized and incorporated into the endoplasmic reticulum (ER) as large precursor proteins [34]. The depletion of sterol can promote the release of mature SREBPs proteins that are transported from ER to the Golgi [31]. The reduction of fat mass, hepatic, and plasma triglycerides was observed in SREBP1c-specific knockout mice [35]. In the present study, the results of subcellular localization and functional prediction implied that SREBP1 and SREBP2 expressions were closely related with metabolism processes in cells. The differences in the expression levels of SREBFs in sheep among different breeds (different fat tail types) may be attributed due to the differences in tail types between the two sheep breeds. The data from the associations between *SREBF1*/*2* expressions and slaughter and tail traits also confirmed this fact. These results further provide new evidence for revealing the genetic mechanism leading to the difference in sheep tail type. The role of transcription factor genes *SREBF1* and *SREBF2* in the phenotype of fat deposition and the relationship with metabolites were shown in Figure 6.

## 5. Conclusions

Our study revealed that the serum concentrations of TC and NEFA in sheep had significant differences between the studied breeds, and most of the indicators showed the dynamic changes with an age-dependent manner. More importantly, the mRNA expression profiling of *SREBF1* and *SREBF2* showed a breed-specific, gender-specific, and temporal and spatial expressions differences. All results implied that *SREBF1*/*2* played a crucial role in lipid metabolism regulation during growth and development of two fat-tailed sheep, correspondingly, lipid metabolism regulation pattern in STH and GLT may be distinguished. This study revealed a new phenotypic basis for the genetic mechanism in lipid metabolism and fat deposition that caused differences in ovine tail types, which also provided a novel insight for meat quality improvement.

## Figures and Tables

**Table 1:** Correlation coefficients between *SREBF1* mRNA expression in different tissues and slaughter and tail traits in sheep.

Breed	Traits	Tissues							
		TA	GO	SC	SO	PR	RP	MT	LV
Guangling Large Tailed sheep (GLT)	TL	−0.03	0.67 *	0.05	0.45	−0.12	0.06	0.47	0.67 *
	TW	0.41	0.26	0.43	0.01	−0.02	0.34	0.29	0.43
	ATW	0.65 *	0.21	0.55	0.24	0.29	0.31	0.27	0.43
	RTW	0.65 *	0.45	0.35	0.45	0.25	0.52	0.60	0.68 *
	BW	0.10	−0.30	0.28	−0.08	0.11	−0.09	−0.16	−0.12
	CW	0.26	−0.22	0.43	−0.16	0.11	−0.19	−0.27	−0.10
	DP	0.39	−0.02	0.51	−0.19	0.14	−0.28	−0.43	−0.01
Small Tailed Han sheep (STH)	TL	0.70	−0.34	0.45	−0.35	−0.22	0.30	0.31	−0.21
	TW	0.38	0.10	0.23	0.02	0.21	0.03	−0.20	0.23
	BW	0.05	0.42	0.04	0.12	0.71 *	−0.17	0.14	0.64
	CW	−0.01	0.38	−0.01	0.02	0.73 *	−0.28	0.15	0.57
	DP	−0.36	−0.45	−0.41	−0.60	−0.15	−0.63	0.02	−0.42

TL = tail length; TW = tail width; ATW = absolute tail fat weight; RTW = relative tail fat weight; BW = body weight; CW = carcass weight; DP = dressing percentage; TA = tail fat; GO = great omental fat; SC = subcutaneous fat; SO = small omental fat; PR = perirenal fat; RP = retroperitoneal fat; MT = mesenteric fat; LV = liver. * *p* < 0.05.

**Table 2:** Correlation coefficients between *SREBF2* mRNA expression in different tissues and slaughter and tail traits in sheep.

Breed	Traits	Tissues							
		TA	GO	SC	SO	PR	RP	MT	LV
Guangling Large Tailed sheep (GLT)	TL	−0.10	0.03	−0.52	−0.01	−0.45	−0.36	−0.29	−0.69 *
	TW	−0.16	−0.50	−0.61 *	−0.11	−0.53	−0.19	−0.27	−0.77 *
	ATW	−0.34	−0.61 *	−0.44	−0.31	−0.25	−0.21	−0.39	−0.72 *
	RTW	−0.49	−0.36	−0.17	−0.24	−0.07	−0.02	−0.21	−0.55
	BW	0.08	−0.46	−0.56	−0.31	−0.25	−0.22	−0.33	−0.30
	CW	0.07	−0.59 *	−0.51	−0.24	−0.22	−0.30	−0.43	−0.50
	DP	−0.03	−0.57	−0.30	−0.39	−0.18	−0.43	−0.41	−0.59
Small Tailed Han sheep (STH)	TL	0.48	0.49	0.30	−0.07	0.63	−0.09	0.04	−0.20
	TW	0.19	0.28	0.37	0.04	0.55	0.08	0.37	0.10
	BW	−0.44	0.27	0.58	0.53	0.16	−0.43	−0.05	0.47
	CW	−0.39	0.23	0.66 *	0.53	0.24	−0.42	−0.04	0.48
	DP	0.36	−0.27	0.30	−0.14	0.08	0.21	0.07	−0.12

TL = tail length; TW = tail width; ATW = absolute tail fat weight; RTW = relative tail fat weight; BW = body weight; CW = carcass weight; DP = dressing percentage; TA = tail fat; GO = great omental fat; SC = subcutaneous fat; SO = small omental fat; PR = perirenal fat; RP = retroperitoneal fat; MT = mesenteric fat; LV = liver. * *p* < 0.05.

## Supplementary Materials

The following are available online at https://www.mdpi.com/2076-2615/10/8/1317/s1, Table S1: Analysis of variance in serum biochemical indexes of two fat-tailed sheep (mmol/L), Table S2: Abundance of ovine SREBF1 mRNA expression, Table S3: Correlation coefficients of SREBF1 mRNA expression in adipose tissues between two fat-tailed sheep, Table S4: Abundance of ovine SREBF2 mRNA expression, Table S5: Correlation coefficients of SREBF2 mRNA expression in adipose tissues between two fat-tailed sheep.
