# Supplementary material for: Regulatory Roles of SREBF1 and SREBF2 in Lipid Metabolism and Deposition in Two Chinese Representative Fat-Tailed Sheep Breeds
Source: Animals (Basel). 2020 Jul 30;10(8):1317. doi: 10.3390/ani10081317 (PMC7460493; doi:10.3390/ani10081317)
Supplement: Supplementary file 1 [file animals-10-01317-s001.pdf]

animals

MDPI

Article

Regulatory Roles of *SREBF1* and *SREBF2* in Lipid Metabolism and Deposition in Two Chinese Representative Fat-tailed Sheep Breeds

Chen Liang[1], Liying Qiao[1], Yongli Han[2], Jianhua Liu[1], Jianhai Zhang[2] and Wenzhong Liu[1,*]

[1] College of Animal Science, Shanxi Agricultural University, Taigu, Shanxi, People's Republic of China, 030801.

[2] College of Veterinary Medicine, Shanxi Agricultural University, Taigu, Shanxi, People's Republic of China, 030801.

*Correspondence: tglwzyc@163.com; Tel.: +086-354-6288227

Supplementary Material

Table S1. Analysis of variance in serum biochemical indexes of two fat-tailed sheep (mmol/L)

Factor	Level	TG	HDLC	LDLC	TC	NEFA
Breed	GLT	0.09±0.02^{a}	0.66±0.09^{a}	1.01±0.23^{a}	1.72±0.13^{b}	0.34±0.04^{a}
	STH	0.10±0.02^{a}	0.70±0.07^{a}	1.09±0.14^{a}	2.03±0.08^{a}	0.21±0.03^{b}
Gender	Male	0.10±0.02^{a}	0.66±0.09^{a}	1.32±0.24^{a}	1.99±0.11^{a}	0.23±0.03^{b}
	Female	0.09±0.01^{a}	0.70±0.07^{a}	0.79±0.13^{a}	1.77±0.10^{a}	0.33±0.03^{a}
Month of age	4	0.10±0.02^{a}	0.64±0.12^{a}	0.65±0.27^{a}	1.50±0.13^{a}	0.46±0.05^{a}
	6	0.08±0.02^{a}	0.53±0.13^{a}	1.02±0.20^{a}	1.74±0.14^{a}	0.23±0.04^{b}
	8	0.07±0.04^{a}	0.64±0.14^{a}	1.07±0.34^{a}	1.49±0.19^{a}	0.24±0.05^{b}
	10	0.06±0.02^{a}	0.77±0.13^{a}	1.12±0.22^{a}	1.86±0.14^{a}	0.29±0.04^{ab}
	12	0.02±0.05^{a}	0.44±0.17^{a}	1.31±0.44^{a}	1.84±0.20^{a}	0.20±0.07^{b}

GLT = Guangling Large Tailed sheep; STH = Small Tailed Han sheep; TG = triglyceride; HDLC = high-density lipoprotein cholesterol; LDLC = low-density lipoprotein cholesterol; TC = total cholesterol; NEFA = non-essential fatty acid. The values with different lowercase superscripts within the same factor indicate a significant difference (P<0.05).

Table S2. Abundance of ovine *SREBF1* mRNA expression

Factor	Level	Abundance of mRNA	Factor	Level	Abundance of mRNA
Breed	Guangling Large Tailed sheep	1.773±0.125^{b}	Gender	Male	1.916±0.148^{a}
	Small Tailed Han sheep	2.160±0.149^{a}		Female	2.017±0.127^{a}
Tissue	Tail fat (TA)	1.856±0.257^{b}	Month of age		
	Great omental fat (GO)	1.803±0.261^{b}		4	2.027±0.224^{abc}
	Subcutaneous fat (SC)	1.849±0.259^{b}		6	1.605±0.216^{bc}
	Small omental fat (SO)	1.721±0.261^{b}		8	1.439±0.218^{c}
	Perirenal fat (PR)	1.803±0.260^{b}		10	2.351±0.215^{ab}
	Retroperitoneal fat (RP)	1.119±0.264^{b}		12	2.417±0.320^{a}
	Mesenteric fat (MT)	1.034±0.265^{b}			
	Liver (LV)	4.547±0.256^{a}			

The values with different lowercase superscripts within the same factor indicate a significant difference ($P<0.05$).

Table S3. Correlation coefficients of *SREBF1* mRNA expression between tissues in two fat-tailed sheep

Tissues	TA	GO	SC	SO	PR	RP	MT	LV
TA	1	-0.331	**0.852***	-0.068	-0.346	0.348	0.516	0.340
GO	-0.160	1	-0.279	0.516	**0.915***	-0.521	-0.363	0.665
SC	0.000	0.074	1	0.300	-0.039	0.416	0.322	0.075
SO	0.138	0.422	0.132	1	**0.799***	-0.046	-0.149	0.653
PR	0.376	-0.256	0.117	0.297	1	-0.259	0.070	0.435
RP	0.165	0.315	0.306	0.021	0.301	1	0.314	-0.423
MT	-0.051	0.511	0.080	0.666	0.224	0.425	1	-0.382
LV	0.223	**0.854****	0.121	0.718	-0.208	0.456	0.617	1

TA = tail fat; GO = great omental fat; SC = subcutaneous fat; SO = small omental fat; PR = perirenal fat; RP = retroperitoneal fat; MT = mesenteric fat; LV = liver. The lower-left corner belongs to Guangling Large Tailed sheep, and the value of Small Tailed Han sheep is at upper-right corner. *$P<0.05$，** $P<0.01$

Table S4. Abundance of ovine *SREBF2* mRNA expression

Factor	Level	Abundance of mRNA	Factor	Level	Abundance of mRNA
Breed	Guangling Large Tailed sheep	1.451±0.062^{a}	Gender	Male	1.090±0.074^{b}
	Small Tailed Han sheep	0.975±0.076^{b}		Female	1.336±0.063^{a}
Tissue	Tail fat (TA)	1.047±0.135^{bc}			
	Great omental fat (GO)	1.284±0.129^{b}	Month of age	4	1.367±0.111^{a}
	Subcutaneous fat (SC)	1.277±0.134^{b}		6	1.238±0.107^{a}
	Small omental fat (SO)	1.147±0.134^{b}		8	1.049±0.106^{a}
	Perirenal fat (PR)	1.102±0.142^{b}		10	1.118±0.105^{a}
	Retroperitoneal fat (RP)	1.029±0.135^{bc}		12	1.223±0.166^{a}
	Mesenteric fat (MT)	0.706±0.135^{c}			
	Liver (LV)	2.113±0.128^{a}			

The values with different lowercase superscripts within the same factor indicate a significant difference *P*<0.05).

Table S5. Correlation coefficients of *SREBF2* mRNA expression between tissues in two fat-tailed sheep

Tissues	TA	GO	SC	SO	PR	RP	MT	LV
TA	1	-0.163	0.058	-0.462	0.089	-0.054	0.301	-0.236
GO	0.187	1	0.424	**0.680***	**0.779***	-0.147	-0.163	-0.328
SC	0.149	0.218	1	0.397	**0.709***	-0.327	0.064	0.066
SO	-0.093	0.148	-0.003	1	**0.762***	-0.089	-0.112	0.065
PR	0.026	-0.153	**0.860****	0.052	1	-0.107	-0.102	-0.703
RP	0.475	0.043	-0.060	-0.182	-0.087	1	0.464	-0.344
MT	0.209	0.145	0.568	**0.697***	**0.666***	0.100	1	-0.276
LV	0.107	0.158	**0.672***	0.222	**0.829****	0.112	**0.699***	1

TA = tail fat; GO = great omental fat; SC = subcutaneous fat; SO = small omental fat; PR = perirenal fat; RP = retroperitoneal fat; MT = mesenteric fat; LV = liver. The lower-left corner belongs to Guangling Large Tailed sheep, and the value of Small Tailed Han sheep is at upper-right corner. *$P<0.05$，** $P<0.01$
